# Leiomyosarcoma of the Rectum as a Radiation-Induced Second Malignancy after Cervical Cancer Treatment: Case Report with Review of the Literature

**DOI:** 10.1155/2019/1610653

**Published:** 2019-12-10

**Authors:** Dmytro E. Makhmudov, Olena O. Kolesnik, Natalia N. Lagoda, Maryna O. Volk

**Affiliations:** ^1^Oncocoloproctology Department, National Cancer Institute, Lomonosova str. 33/43, Kyiv 03022, Ukraine; ^2^Department of Pathomorphology, National Cancer Institute, Lomonosova str. 33/43, Kyiv 03022, Ukraine

## Abstract

**Background:**

Incidence of cervical cancer among women of reproductive age still remains significantly high. In regard to prognostic features and risk factors, the standard treatment for most types of cervical cancer represents a combination of surgical treatment and radiation therapy, such as external beam radiation therapy and brachytherapy. Despite significant advances of long-term oncological outcomes, radiation-induced secondary malignancies among cervical cancer survivors are still an issue. Current case report describes an incredibly rare case of radiation-induced leiomyosarcoma of the rectum, which occurred 32 years after cervical cancer treatment.

**Case Presentation:**

A 62-year-old female had a past medical history of FIGO stage IIB cervical cancer (squamous cell carcinoma pT2bN0M0). In 1987, she underwent radical hysterectomy with bilateral iliac lymph node dissection, followed by adjuvant radiation therapy—70 Gy external beam pelvic irradiation followed by 30.5 Gy of brachytherapy. Thirty-two years later, she presented with signs of rectal bleeding. Regarding past medical history, radiologic, endoscopic, and pathologic data, the patient was initially diagnosed with a malignant nonepithelial lower rectal tumor of the unknown origin and staged as mrT3a mrN0 cM0. Total mesorectal excision with complete mesocolic excision and central vascular ligation (CME/CVL) carried by an open approach was carried out. In an attempt to identify the tissue of origin, an immunohistochemistry assay had been performed. Tumor cells showed a high rate of mitotic activity with a 45% rate of Ki-67 expression, positive reaction for desmin, and SMA in all samples. Negative reaction for CD117 and S100 was observed. As a conclusion, the immunophenotype was identified as a grade 3 leiomyosarcoma (ISD-code 8890/3).

**Conclusions:**

We suggest that up to date, radical surgery with curative intent, as it was performed in our study, is the most evidence-based treatment option for patients with radiation-induced sarcomas of the rectum.

## 1. Background

Primary colorectal sarcomas are an extremely rare group of malignant mesenchymal tumors. They represent up to 0.1% of all primary diagnosed colorectal malignancies [1]. Leiomyosarcomas appear to be the most common histological subtype of primary colorectal sarcomas with incidence up to 90%. More rare of their subtypes are liposarcomas, fibrous histiocytomas, and desmoplastic small cell tumors [2]. Regarding the small number of documented cases and their scattered features, prognostic factors and optimal treatment strategy for primary colorectal sarcomas remain indeterminate. However, pooled data from a majority of recently published case series suggest that primary colorectal sarcomas are characterized by both rapid progression and very poor oncological outcomes with a median of survival ranging from 30 to 53 months and a local recurrence rate of up to 85% [3–6]. Overall five-year survival rate of patients with primary colorectal sarcomas is significantly lower comparing to those with colorectal cancer—43% and 52%, respectively [7]. Tumor grade of primary colorectal sarcomas is the most important prognostic factor for overall five-year survival—38% for high grade and 61% for low grade [8]. Besides, inappropriate staging algorithm and wide use of local excision as a surgical option for primary treatment results in high rates of R1 and R2 resections with a local recurrence rate of 12.7%. Nevertheless, a number of authors suggest that curative surgery should be the main treatment option because data regarding chemotherapy and radiation therapy is either lacking or it is controversial [9, 10]. Colorectal sarcomas as a radiation-induced second malignancy after cervical cancer treatment, on contrary to primary ones, are an exceptionally rare entity. Up to date, only five cases had been described in the literature [11–15]. In our current paper, we provide data on the sixth case of similar malignancy.

## 2. Case Presentation

A 62-year-old female had a past medical history of FIGO stage IIB cervical cancer (squamous cell carcinoma pT2bN0M0). In 1987, she underwent radical hysterectomy with bilateral iliac lymph node dissection, followed by adjuvant radiation therapy—70 Gy external beam pelvic irradiation followed by 30.5 Gy of brachytherapy. Thirty-two years later, she presented with signs of rectal bleeding.

Digital rectal examination revealed a solid exophytic tumor on 5 cm above the anal verge. During rigid proctosigmoidoscopy, a 3 cm anterior wall rectal tumor with irregular margins and swollen mucosa was observed. Additional flexible colonoscopy showed no signs of synchronous colorectal neoplasms. Snare biopsy result showed an undifferentiated malignant nonepithelial tumor of unidentified histological origin. An attempt of immunohistochemistry assay was undertaken unsuccessfully due to an insufficient amount of tissue.

Consecutively, a pelvic MRI with a 1.5 Tl Philips Intera machine had been performed for local staging. MERCURY protocol for high resolution imaging was applied.

**Figure 1:**
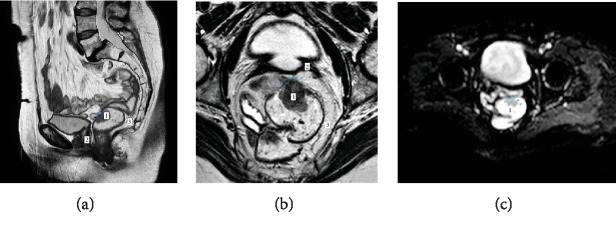
Radiologic data from a high-resolution pelvic MRI. Tumor of the lower rectum (1) situated on the anterior wall was observed on sagittal (a) and axial T2-weighted scans (b) with signs of mesorectal invasion (blue arrowheads) ranging up to 3 mm and no radiologic features of lymph node metastases (3). Vaginal stump and rectovaginal sept (2) had a significantly low intensity MR signal due to their fibrotic transformation. On a series of diffusion-weighted images (c), a significant delay of diffusion together with intense signaling on high b-factors was observed at the level of a tumor (blue arrowheads).

On a series of sagittal and axial MR scans, a lower rectal tumor was observed on 4 cm above the anorectal junction with maximal extent of 30 mm in the greatest dimension. Mesorectal infiltration with maximal depth of 3 mm (mrT_3b_) was also identified. No radiological signs of either mesorectal fascia involvement or extramural vascular invasion (mrCRM^−^ and mrEMVI^−^) had been observed (see Figures 1(a) and 1(b)). Visible mesorectal lymph nodes had homogenous MR signal, smooth external margins, and a regular size, thus showing no radiologic signs of lymph node involvement (mrN_0_). It was noticeable that the stump of the vagina and rectovaginal sept had prominent signs of fibrosis regarding a highly intensive MR signal on T2-weighted images. Malignant tumor features were also observed on a series of diffusion-weighted images (see Figure 1(c)).

A CT scan with intravenous enhancement showed no signs of neither distant metastases neither thoracic or intra-abdominal lymphadenopathy. Upper GI endoscopy and laboratory data of standard blood, serum, and urine counts revealed no signs of pathology.

## 3. Surgical Treatment

**Figure 2:**
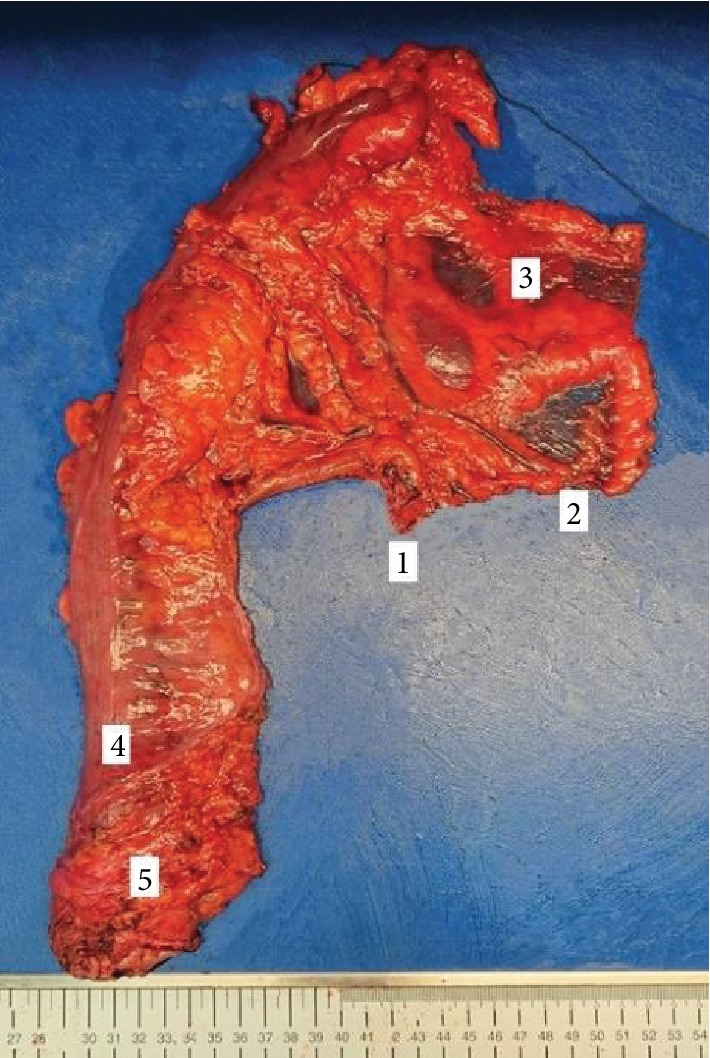
Resected gross specimen. 1: stump of the inferior mesenteric artery, 2: stump of the inferior mesenteric vein, 3: preserved peritoneal window, 4: level of the peritoneal reflection, and 5: mesorectum.

Regarding past medical history, radiologic, endoscopic, and pathologic data, the patient was initially diagnosed with a malignant nonepithelial lower rectal tumor of the unknown origin and staged as mrT3a mrN0 cM0. Being aware of primary colorectal sarcomas, their recurrence and progression patterns and a threatening rate of R1 and R2 resections, an institutional multidisciplinary board suggested curative surgery as a primary treatment option. Total mesorectal excision (TME) with complete mesocolic excision and central vascular ligation (CME/CVL) carried by an open approach was selected as proper extent of surgery. Standardized surgical technique in this case included complete left flexure mobilization, ligation of the inferior mesenteric artery at its origin near the aorta and inferior mesenteric vein just below the pancreatic tail with consecutive total mesorectal excision up to the level of the pelvic diaphragm (see Figure 2). Accurate TME procedure had been exacerbated by severe fibrotic changes of surrounding pelvic tissues due to postoperative changes after iliac lymphadenectomy for cervical cancer and radiation-induced fibrosis as well. Regarding the significant fibrotic transformation of the anterior rectal wall with adjacent tissues, an intraoperative decision was made to avoid reconstruction aware of an estimated high risk of anastomotic leakage. Closure of the pelvic peritoneum was avoided as well.

**Figure 3:**
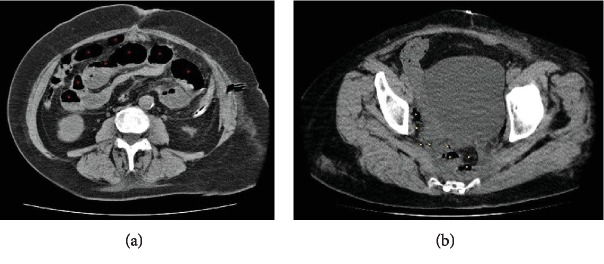
Patient's CT scans on the 6^th^ postoperative day. Multiple intestinal “levels” of gas and fluid throughout the abdominal cavity were observed ((a), red arrowheads). Part of the distal intestinal loop with difference in lumen diameters was situated at the level of the pelvic floor ((b), yellow arrowheads) and was adherent to both rectal and vaginal stumps.

On postoperative day 5, the patient developed such clinical signs as nausea, vomiting, and loss of flatus and stool. Computed tomography of the chest, abdomen, and pelvis revealed multiple “levels” of gas and liquid throughout the small intestine with a breakdown of peroral contrast at the level of distal ileal loop, which was situated at the cavity of minor pelvis, adherent to the stumps of both the rectum and the vagina (see Figure 3). At that level, a noticeable deformation of the intestinal loop with difference of luminal diameters was observed. On contrary, the lumen of a large intestine was collapsed.

**Figure 4:**
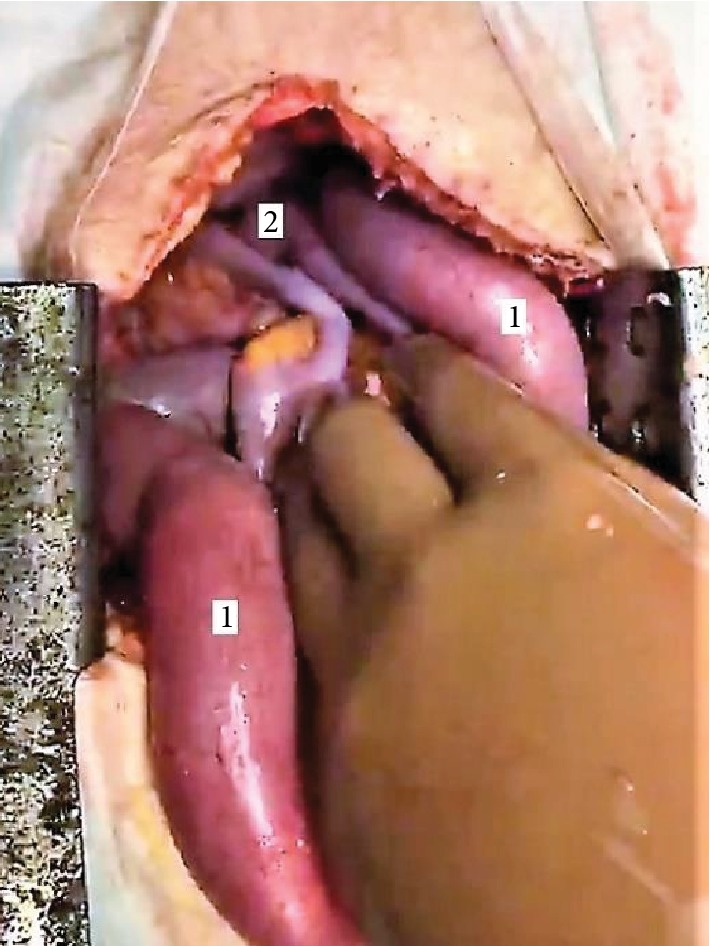
Intraoperative photo during reoperation. Dilated intestinal loops up to the level of distal ileum (1) are observed. The loop of a strangulated bowel (2) was adherent to the stump of the rectum and vagina deep inside the pelvic cavity.

By taking in regard both clinical and radiologic findings, the patient was diagnosed with postoperative ileus and an immediate reoperation had been carried out. During abdominal examination, dilated intestinal loops with block of passage at the level of the pelvic diaphragm were identified (see Figure 4). An adherent bowel wall was fixed between vaginal and rectal stumps as it was previously described on a series of CT scans. By the end of abdominal and pelvic exploration, no additional pathological findings were observed. After detachment of adhesions, the strangulated bowel was delivered back in the abdominal cavity. Closure of the pelvis was performed with mobilized body of the urinary bladder and remnant flaps of pelvic peritoneum. Postoperative period was uneventful within 30 days. The patient was safely discharged on postoperative day 14 after initial surgery. At one year of follow-up, the patient is alive and has no radiological signs of neither local nor distant recurrence.

## 4. Pathologic Findings

**Figure 5:**
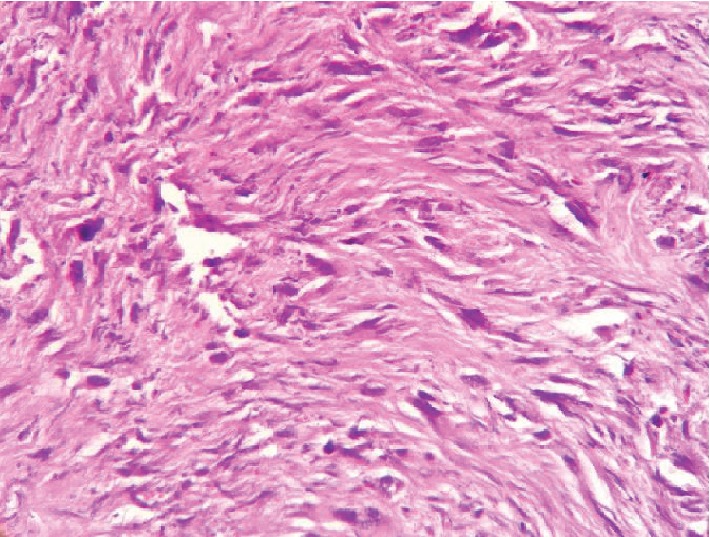
Poorly differentiated malignant mesenchymal tumor. Multiple sites of cellular and nuclear polymorphism are observed. Hematoxylin-eosin staining, ×100.

Macroscopically, the tumor was represented an exophytic homogenous lesion 45 × 25 mm with irregular margins and ulcerated surface covered with clots of fibrin. The tumor originated from the muscular layer of the rectum with concomitant mucosal invasion. Twelve regional lymph nodes were additionally examined. Initial histological appearance revealed a mixture of chaotic cellular and fibrotic vegetations. Among them, a subgroup of heterogeneous polykaryocytes with significant nuclear polymorphism (so called “monster cells”) was identified (see Figure 5). Those and other morphological features corresponded to a malignant low-grade mesenchymal tumor. All examined lymph nodes had no signs of metastases.

**Figure 6:**
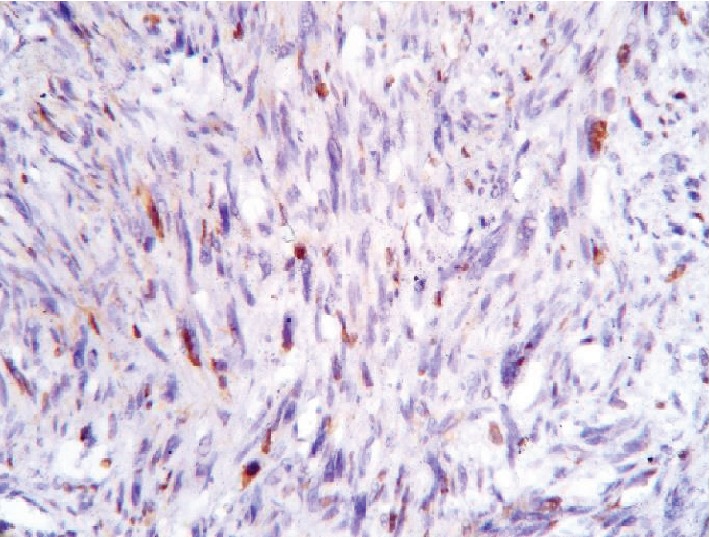
Poorly differentiated malignant mesenchymal tumor. Immunohistochemistry assay with Ki-67 antibodies.

**Figure 7:**
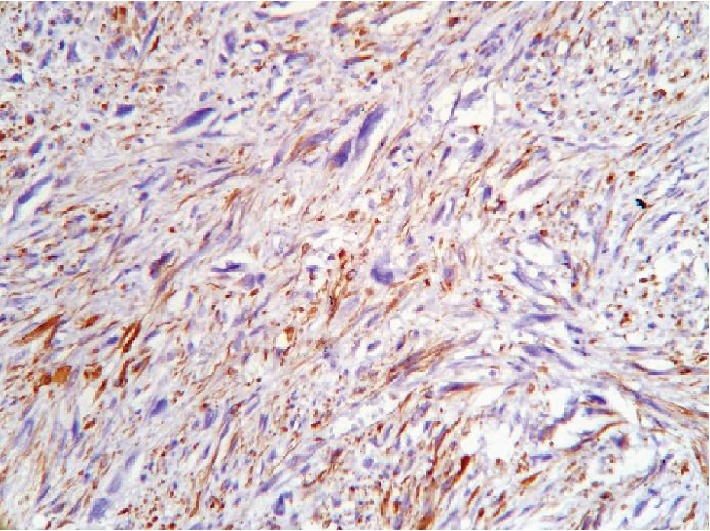
Poorly differentiated malignant mesenchymal tumor. Immunohistochemistry assay with desmin antibodies. Positive reaction in tumor cells observed.

**Figure 8:**
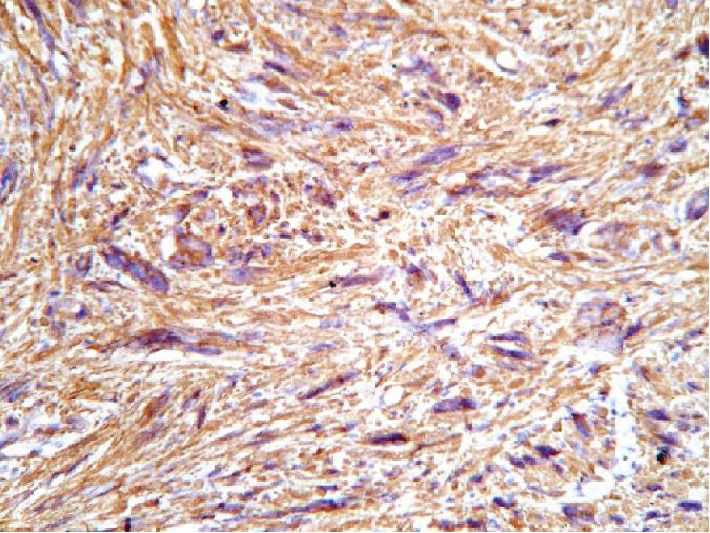
Poorly differentiated malignant mesenchymal tumor. Immunohistochemistry assay with SMA antibodies. Positive reaction in tumor cells observed.

**Figure 9:**
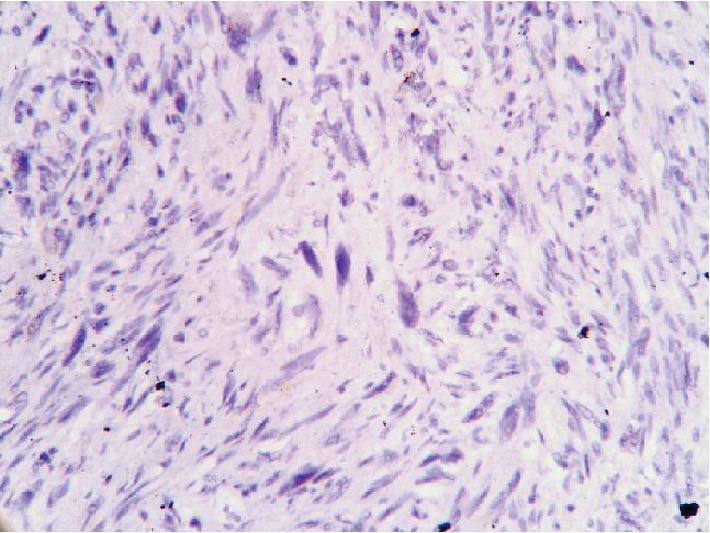
Poorly differentiated malignant mesenchymal tumor. Immunohistochemistry assay with CD117 antibodies. Negative reaction in tumor cells observed.

**Figure 10:**
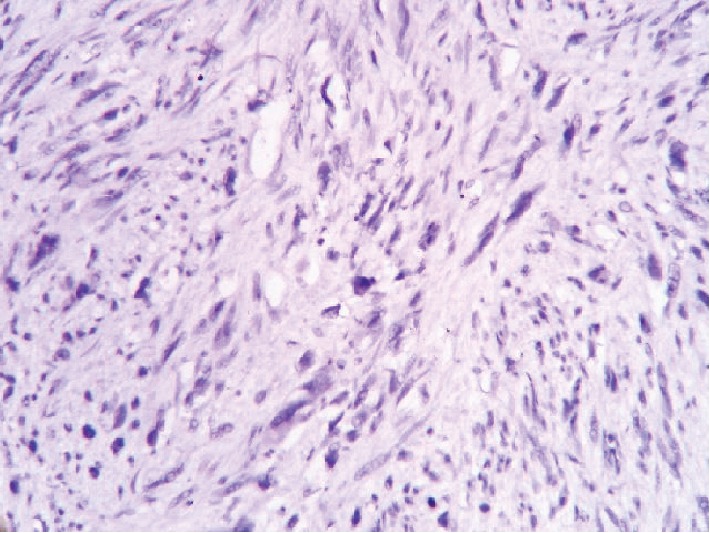
Poorly differentiated malignant mesenchymal tumor. Immunohistochemistry assay with S100 antibodies. Negative reaction in tumor cells observed.

In an attempt to identify the tissue of origin for a current tumor, an immunohistochemistry assay had been carried out. A panel of Ki-67, CD117, S100, smooth muscle actin (SMA), and desmin was used to conduct a differential diagnosis between leiomyosarcoma and gastrointestinal stromal tumor. Tumor cells showed a high rate of mitotic activity with a 45% rate of Ki-67 expression (see Figure 6). There was a positive reaction for desmin and SMA in all samples (see Figures 7 and 8). On contrary, all samples had negative reaction for CD117 and S100 (see Figures 9 and 10). As a conclusion, the observed immunophenotype was identified as a grade 3 leiomyosarcoma (ISD-code 8890/3).

## 5. Discussion

In the current paper, we present the sixth case of radiation-induced leiomyosarcoma of the rectum which developed 32 years after completion of cervical cancer treatment. Throughout the last decades, significant success was achieved in the treatment of patients with pelvic malignancies, especially those who require either adjuvant or neoadjuvant radiation therapy. As a consequence of overall survival improvement, the problem of metachronous secondary malignancies among those patients who were exposed to radiation had arisen. According to the data of the National Cancer Institute of USA, over 40% of all patients diagnosed with primary malignancy will have to undergo radiation therapy [16]. Most of primary malignancies which require radiation therapy as a treatment standard are breast, prostate, cervical, rectal, and urinary bladder cancer. Among long-term cancer survivors exposed to radiation, 16% are at risk of developing a secondary malignancy. Recent data from a SEER database suggests that 6–9.9% of all patients who underwent radiation therapy for prostate cancer are at a 34% risk of developing secondary lung, rectal, or urinary bladder cancer. Majority of those patients develop secondary malignancies mostly after 10 years from treatment discontinuation [17, 18].

Cervical cancer remains one of the leading cause of cancer-related morbidity and mortality among females [19]. It is known that only surgical treatment can be appropriate only in a relatively small subgroup of patients, particularly among those who were initially diagnosed with FIGO stage IA1 (T1a1 by TNM) or less, i.e., with a depth of stromal invasion up to 3 mm and lateral spread up to 7 mm [20]. Recent NCCN guidelines suggest adjuvant radiation therapy for FIGO stage IA1 patients with a number of adverse risk features found at pathological examination, such as positive lymph nodes, parametrium invasion, or positive resection margins [21]. A standard approach for cervical cancer radiation therapy includes combination of external beam radiation therapy and brachytherapy. Overall radiation dosage depends on the range of primary tumor spread—in cases of tumors below 40 mm in maximum dimension, the overall dosage consists of 80 Gy and ≥85 Gy if above 40 mm.

In cases of cervical cancer, the radiation field includes parametrium, sacrouterine ligaments, 3 cm of upper third of the vagina below the tumor, and presacral, obturator, external, and internal iliac lymph nodes. By taking in regard all the anatomical relations of the cervix, it becomes clear that the most critical radiation sites are represented by the vagina, the rectum, and the urinary bladder. In comparison, bony pelvis structures receive significantly lesser radiation dosages.

A successful combination of curative surgery and highly precise modalities of radiation therapy in either adjuvant or neoadjuvant regimens had given a unique opportunity to improve cervical cancer patient's overall and disease-free survival rates like never before. Hence, by dropping out of a cancer recurrence group, those patients consequently enter another one—a risk group of developing a secondary malignancy. The first population-based study which highlighted a relevance between previous exposure to pelvic irradiation and a risk of developing a second malignancy was presented by Boice et al., in 1984 [22]. The study included more than 95,000 patients after pelvic radiation therapy for cervical cancer within a 30-year study period. This population was compared with more than 99,000 patients who underwent only curative surgery. Throughout the study period, 3324 (3.5%) secondary radiation-induced malignancies had been observed, of which 1622 (1.7%) were located at the organs covered by the irradiation field. The main risk factor of secondary malignancy development was the age under 30 years at primary diagnosis of cervical cancer. Urinary bladder (4.5), vagina (2.7), stomach (2.1), and hematopoietic tissue (2.5) had the highest relative risk for secondary malignancy. It was noticeable that the rectum had the lowest relative risk among other pelvic organs (1.8). Other data from a study of Samerdokiene et al. suggest that secondary radiation-induced malignancies occur in 5.3% of cervical patients after a combination of external beam and brachytherapy. Among them, rectal malignancies consisted only 8.6% [23]. A population study of 37,757 patients based on a data from SEER database demonstrated a 2.6-fold increase in a number of secondary malignancies among those cervical cancer patients who underwent pelvic radiation therapy comparing to surgery alone [24]. Nevertheless, the authors acknowledged that metachronous bronchopulmonary, esophageal, and oropharyngeal cancer were mostly caused by continuous smoking and vaginal, vulval, and anal canal cancer—by HPV infection rather than by previous radiation exposure. Similar retrospective population-based data concerning a consistent pattern of an increased secondary malignancy rate among cervical cancer survivors was obtained from the Netherlands and Taiwan [25, 26]. Even though all secondary colon, rectal, and anal malignancies in those studies were united in one subgroup, their relative risk ratio still remained one of the lowest. A study by Ohno et al., based on a pooled data from 2167 patients after a combination of external beam and brachytherapy for cervical cancer, reports a 9.7% rate of secondary radiation-induced malignancies with 19% of them related to adjacent irradiated organs [27]. Soft tissue and bone sarcomas (22.0), leukemias (3.1), and urinary bladder cancer (2.2) had the greatest relative risk rates. On the other hand, rectal cancer, hepatocellular carcinoma, and gastric cancer (1.0, 1.2 and, respectively) possessed the lowest relative risk rates. Lim et al. came up with a data of 72,805 invasive cervical cancer patients after pelvic irradiation within a study period of 7.34 years [28]. A 3.68% rate (2678 cases) of secondary radiation-induced malignancies with similar relative risk patterns—vagina (9.36), soft tissues and bones (2.7), vulva (2.58), and anus and anal canal (2.42)—was observed. It is noticeable that among the 35 sites of secondary malignancy occurrence, rectal cancer had the lowest relative risk of 0.74. Correlation between previously treated cervical cancer secondary colorectal cancer had been recently highlighted in a paper of Rodriguez et al. [29]. After 35 years of follow-up, an estimated risk of colon cancer was 2.5% in a surgery alone group and 6.5% in a radiation therapy group. For rectal cancer, the difference was more significant—0.8% and 3.7%, respectively. Regression analysis survival model demonstrated a significant increase of relative risk for colon cancer development after 8 years and rectal cancer—after 15 years of follow-up. The authors suggest that 8 years should be a cutoff edge for colorectal cancer screening among cervical cancer survivors.

First studies about the influence of ionizing radiation on the development of malignancies were provided after a nuclear attack in Japan at 1945. Three main issues had been discovered then: tissues with higher proliferative index (i.e., epithelial and hematopoietic) are mostly affected; very small number of radiation-induced sarcomas and a ratio of 8%/1 Gray, which is a distribution of those who shall develop a malignancy among one hundred people exposed to an irradiation dosage of 1 Gy [30]. Mechanisms and conditions for radiation sarcoma development were firstly described by Cahan et al. in 1948 [31]. It was noticed that the occurrence of pathologically confirmed secondary sarcomas is related to an irradiation field previously exposed to a dosage of at least 50 Gy. However, the only uncertainty was timing of occurrence which could range from months to decades. Despite meeting all of Cahan's criteria, radiation-induced sarcomas in cervical cancer patients after pelvic radiation therapy are a very rare entity. Among all primary diagnosed soft tissue sarcomas, a range of 0.03% to 5.5% could be considered as radiation induced [32, 33].

According to the largest published series of Cha et al., only 125 (2.5%) out of 4884 primary soft tissue sarcomas satisfied Cahan's criteria and were considered as radiation induced [32]. Vast majority of those patients previously underwent radiation therapy for breast cancer (29%), lymphomas (16%), and prostate cancer (14%) with thoracic cavity, thoracic wall, extremities, head and neck as a tumor site. Pathological features of leiomyosarcomas were identified only in 12% of radiation-induced sarcomas. However, there was absolutely no data regarding a radiation-induced leiomyosarcoma after previous pelvic radiation therapy with intra-abdominal or either intrapelvic localization.

Remarkably, but up to date, there were only 5 published case reports of a radiation-induced leiomyosarcoma of the rectum in a patient after previous radiation therapy for cervical cancer (see Table 1). On the contrary, one of the largest recent studies by Thiels et al. evaluates a series of 433 primary colorectal sarcomas discovered within a 14-year observation period [8]. Among the 29.3% of patients with the rectum as a primary tumor site, only 57.5% had leiomyosarcomas.

Although radiation-induced leiomyosarcomas of the rectum appear to be an extremely rare type of secondary malignancies, there is an emerging data suggesting an intrinsically different sequence of molecular events responsible for their development and occurrence comparing to primary sarcomas [14]. Gonin-Laurent et al. recognize a mutation of ТР53 and RB1 genes as a key molecular event in the development of radiation-induced sarcomas. Consecutive mutation of a TP53 gene was identified in 58% of radiation-induced sarcomas and was related to deletion of other oncogenes in 52% [34, 35]. It was also mentioned that mutation of a TP53 gene led to inactivation of RB1, which showed no signs of genetic alterations [35]. Hyperexpression of р53 is recognized as a specific pathogenic route for radiation-induced sarcoma development as well. Taubert et al. identified a р53 mutation in 9 out of 11 radiation-induced sarcoma cases [36]. Finally, a study of Nakanishi et al. revealed a р53 mutation pattern in a series of 14 secondary radiation-induced soft tissue sarcomas in patients who previously underwent pelvic radiation therapy for cervical cancer [37]. Real-time polymerase chain reaction detected polymorphism of р53 gene with an 88% rate of mutations in exons 5, 7, 8, 12, and 18. However, concomitant changes in the primary structure of a p53 protein were observed only in 31%. The authors conclude that such a biological mislead might be a reason for a long latency period between radiation exposure and clinical manifestation of secondary soft tissue sarcomas.

## 6. Conclusions

Among all radiation-induced malignancies affecting long-term cervical cancer survivors, soft tissue sarcomas occur quite frequently. However, radiation-induced leiomyosarcoma of the rectum represents a remarkably rare case. Although primary colorectal sarcomas represent a very small subgroup of malignant mesenchymal tumors, the data regarding its proper treatment is lacking. On contrary to primary ones, radiation-induced sarcomas possess a number of unique molecular features which make them biologically different. We suggest that up to date, radical surgery with curative intent, as it was performed in our study, is the most evidence-based treatment option for patients with radiation-induced sarcomas of the rectum. Fibrotic changes in the pelvis as a consequence of previous external beam and brachytherapy may significantly jeopardise the completion of a TME procedure. That is why a two-step surgical strategy with delayed reconstruction should be taken in regard.

## Figures and Tables

**Table 1:** Pooled data of all identified case reports of radiation-induced leiomyosarcoma of the rectum after pelvic radiation therapy for cervical cancer.

Authors	Year of publication	Patient's age at diagnosis	Overall irradiation dosage (Gy)	Radiation modality	Time to diagnosis of a secondary malignancy (years)	Selected treatment strategy
Drumea et al. [11]	1993	62	70	40 Gy EBRT^1^+30 Gy BT^2^	17	Intra-abdominal resection of the rectum
Caporale et al. [12]	2003	N/A	N/A	N/A	N/A	N/A
Basu et al. [13]	2012	79	N/A	N/A	26	Intra-abdominal resection of the rectum
Garcia-Ortega et al. [14]	2018	58	85	50 Gy EBRT+35 Gy BT	8	Sigmostomy+chemotherapy+pelvic exenteration+vulvectomy
Jayakumar et al. [15]	2015	58	N/A	N/A	15	Local excision
Current case report	2019	62	100.5	70 Gy EBRT+30.5 Gy BT	32	ТМЕ+CME/CVL

^1^EBRT: external beam radiation therapy. ^2^BT: brachytherapy.
